# A New Approach for Determining Phase Response Curves Reveals that Purkinje Cells Can Act as Perfect Integrators

**DOI:** 10.1371/journal.pcbi.1000768

**Published:** 2010-04-29

**Authors:** Elena Phoka, Hermann Cuntz, Arnd Roth, Michael Häusser

**Affiliations:** 1 Wolfson Institute for Biomedical Research and Department of Neuroscience, Physiology and Pharmacology, University College London, London, United Kingdom; 2 Department of Bioengineering, Imperial College London, London, United Kingdom; École Normale Supérieure, College de France, CNRS, France

## Abstract

Cerebellar Purkinje cells display complex intrinsic dynamics. They fire spontaneously, exhibit bistability, and via mutual network interactions are involved in the generation of high frequency oscillations and travelling waves of activity. To probe the dynamical properties of Purkinje cells we measured their phase response curves (PRCs). PRCs quantify the change in spike phase caused by a stimulus as a function of its temporal position within the interspike interval, and are widely used to predict neuronal responses to more complex stimulus patterns. Significant variability in the interspike interval during spontaneous firing can lead to PRCs with a low signal-to-noise ratio, requiring averaging over thousands of trials. We show using electrophysiological experiments and simulations that the PRC calculated in the traditional way by sampling the interspike interval with brief current pulses is biased. We introduce a corrected approach for calculating PRCs which eliminates this bias. Using our new approach, we show that Purkinje cell PRCs change qualitatively depending on the firing frequency of the cell. At high firing rates, Purkinje cells exhibit single-peaked, or monophasic PRCs. Surprisingly, at low firing rates, Purkinje cell PRCs are largely independent of phase, resembling PRCs of ideal non-leaky integrate-and-fire neurons. These results indicate that Purkinje cells can act as perfect integrators at low firing rates, and that the integration mode of Purkinje cells depends on their firing rate.

## Author Summary

By observing how brief current pulses injected at different times between spikes change the phase of spiking of a neuron (and thus obtaining the so-called phase response curve), it should be possible to predict a full spike train in response to more complex stimulation patterns. When we applied this traditional protocol to obtain phase response curves in cerebellar Purkinje cells in the presence of noise, we observed a triangular region devoid of data points near the end of the spiking cycle. This “Bermuda Triangle” revealed a flaw in the classical method for constructing phase response curves. We developed a new approach to eliminate this flaw and used it to construct phase response curves of Purkinje cells over a range of spiking rates. Surprisingly, at low firing rates, phase changes were independent of the phase of the injected current pulses, implying that the Purkinje cell is a perfect integrator under these conditions. This mechanism has not yet been described in other cell types and may be crucial for the information processing capabilities of these neurons.

## Introduction

Cerebellar Purkinje cells exhibit a wide range of dynamical phenomena. They are intrinsic neural oscillators, firing spontaneously and highly rhythmically in the absence of synaptic input, at a rate of 10–180 Hz [1]–[5]. They also exhibit intrinsic bistability [2], [3], which influences their responses to sensory stimulation [3]. In addition, interactions between spontaneously firing Purkinje cells can result in waves of activity travelling down the cerebellar folia [4], or in high frequency oscillations [6], which may contribute to the generation of precise temporal patterns in the cerebellar network [7]. Hence, the firing of Purkinje cells is highly time- and state-dependent, and thus they represent excellent targets for dynamical systems analysis.

The phase response curve (PRC; [8]–[12]) is a powerful tool to study neuronal dynamics at the cellular level. The PRC describes the effect of a brief perturbation on the firing phase of a neuron, and can be used to predict the response of a neuron to more complex stimulation patterns [8]–[12]. The shape of the PRC is linked to the type of neuronal excitability [13], [14], to oscillatory stability [15] and to network synchronization properties [16]–[19]. Studying Purkinje cell PRCs is therefore an essential step to explore their dynamic repertoire, probe their biophysical mechanisms, and to construct models of Purkinje cells to determine their role in information processing at the network level.

PRCs can be obtained by directly perturbing the membrane potential by short (infinitesimal) square current pulses [8]–[12] or synaptic conductance pulses [12], [19]–[22], and via indirect methods [23]–[25]. Using the direct method, infinitesimal PRCs are obtained by repeatedly injecting brief current pulses while neurons are firing action potentials (APs). Phase and phase perturbation are measured by using the AP immediately preceding the current pulse as a reference, and we refer to these PRCs as “traditional” PRCs throughout this paper. We show using electrophysiological experiments and in simulations that the interspike interval variability present in Purkinje cells introduces a systematic bias in this traditional calculation of the PRC. The bias results from loss of causality caused by the jitter of the APs surrounding the current pulse, and gives rise to an empty triangular region in the PRC plot, which we call the “Bermuda Triangle”. We introduce a method for calculating the PRC which corrects for this bias by using all spikes in the spike train as a reference, one at a time. We refer to PRCs obtained by this method as “corrected” PRCs. Note that in our study both “traditional” and “corrected” PRCs are calculated using the same experimental data: perturbation of the firing of Purkinje cells with brief square current pulses. Using the corrected method we show that the shape of the Purkinje cell PRC changes fundamentally depending on the firing rate of the neuron.

## Results

### A bias in the traditional method for calculating PRCs

**Figure 1:**
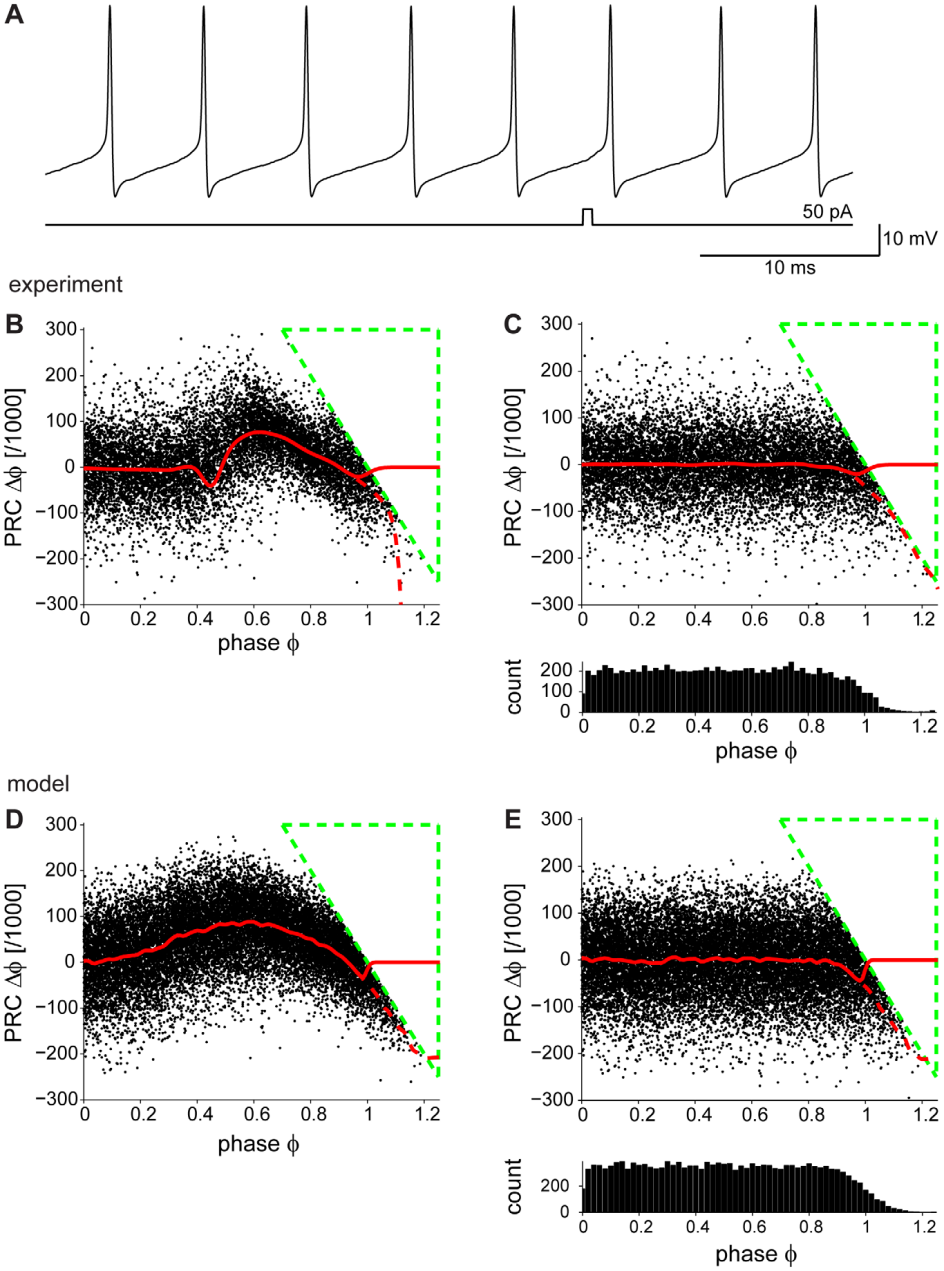
Purkinje cell PRCs determined using the traditional method. (A) Whole-cell patch-clamp recording of a spontaneously firing Purkinje cell. The injected current pulse (0.5 ms) is shown below. (B) Experimentally determined Purkinje cell PRC from the same cell as in (A) (mean firing rate 180 Hz) showing a triangular region (“Bermuda Triangle”, dashed green lines) devoid of points, creating a bias at late phases of the PRC (dashed red line). Deleting the points at phases>1 does not remove the bias (solid red line). (C) Control PRC calculated with no current pulse injection (cPRC) is flat for most of the phases, but the “Bermuda Triangle” and the negative bias at the late phases are still present. The panel below shows the phase histogram, which is inhomogeneous with smaller probabilities of sampling the ISI in the late phases. (D) When noise is introduced in a Purkinje neuron model (firing at its spontaneous firing rate of 27 Hz), the “Bermuda Triangle” and the bias at the late phases are reproduced. (E) Control PRC for the model neuron, calculated in the absence of current pulse injection. It is flat for most of the phases, but the “Bermuda triangle” and the negative deflection at the late phases are reproduced. The phase histogram below is inhomogeneous, with smaller probability of sampling the ISI in the late phases in the model.

Somatic whole-cell patch-clamp recordings were made in current-clamp mode from spontaneously firing Purkinje cells in mouse cerebellar slices. To construct PRCs, a single brief depolarizing current pulse (amplitude, 0.05 nA; duration, 0.5 ms) was injected after a 100–150 ms baseline period (see Fig. 1A). Repeating this protocol many times should result in a homogenous sampling of phase space in spontaneously spiking neurons. The resulting change in interspike interval (ISI) relative to the mean ISI corresponds to the PRC value denoted by 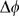. Plotting, for each trial, 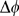 as a function of the phase, 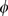, at which the pulse arrived shows the overall ISI shortening corresponding to a positive PRC (Fig. 1B, neuron firing at 180 Hz; see Materials and Methods). Three observations can be made. First, at late phases there is a triangular region entirely void of data points (outlined in green) which we call the “Bermuda Triangle”. This causes a negative bias of the running average at late phases (Fig. 1B, dashed red line). Second, the intrinsic variability in the ISI [1] of spontaneously firing Purkinje cells acts as a source of noise, giving rise to data points with 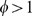. However, removing all points beyond 1 does not eliminate the negative bias (Fig. 1B, solid red line). Finally, many trials (typically more than 5000) were required to calculate the Purkinje cell PRC reliably, while PRCs in other cell types are normally obtained from 100–200 trials [8], [15]. The ISI variability in Purkinje cells [1] results in PRCs with low signal-to-noise ratio, increasing the bias at late phases and leading to a miscalculation of the PRC when this traditional method is used. Thus, a robust and unbiased method for the calculation of Purkinje cell PRCs in the presence of noise is required.

To better understand the negative deflection of the PRC at late phases, a control PRC (cPRC; see Materials and Methods) was calculated from the unperturbed voltage traces prior to the current pulse. The cPRC should be zero throughout all phases. However, the negative bias of the PRC at the late phases persisted in the cPRC (Fig. 1C). We conclude that it is not the result of the brief current pulse injection. Rather, it results from the inhomogeneous sampling of the phase in the presence of noise. Indeed, the phase histogram (Fig. 1C, lower panel) indicates that late phases are sampled less frequently.

To reproduce the effect of noise, PRCs were obtained from a Purkinje cell model [26] in which Gaussian current noise was added to reproduce the irregularity of real Purkinje cell spiking (example model neuron firing at its spontaneous firing rate of 27 Hz; see Materials and Methods). The model PRC exhibited the same negative deflection at late phases as observed in the experimental PRC (Fig. 1D, dashed red line). As before, removing all points for which the phase exceeds 1 did not eliminate the negative deflection (Fig. 1D, solid red line). Similarly, the cPRC in the model exhibited the same negative bias and the same inhomogeneous phase distribution (Fig. 1E) as the experimental cPRC. Therefore, the negative bias at late phases is a general feature of the traditional method for calculating PRCs, and must be due to the intrinsic ISI variability.

**Figure 2:**
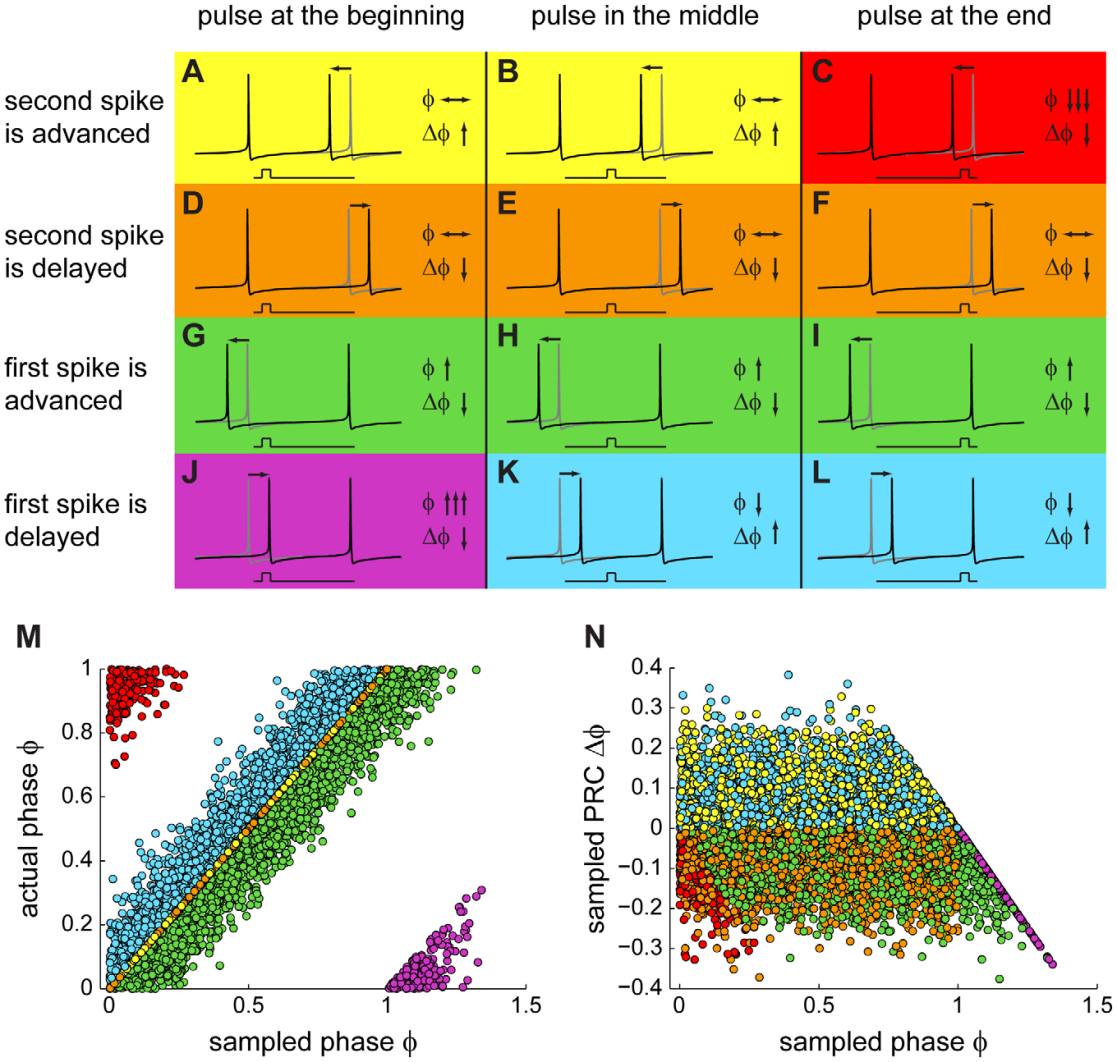
Interspike interval variability causes a bias in the traditional method to calculate PRCs. Illustrations of the changes in 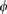 and 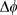 (arrows) introduced by noise, in the form of jittering of the spikes (black lines) surrounding the current pulse, one at a time; as compared to the deterministic case (gray lines). Noise in the PRC plots depends on the position of the pulse within the ISI: (A, D, G, J) the pulse arrives near the beginning of the ISI; (B, E, H, K) the pulse arrives near the middle of the ISI; (C, F, I, L) the pulse arrives near the end of the ISI. The color coding groups the different scenarios according to the effect of the noise on 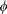 and 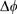. For example, green indicates those scenarios in which noise gave rise to an increase in 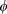 and 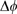 as compared to the deterministic case. (M) Actual phase plotted against the sampled phase (resulting from jittering the spikes around a mean). The color coding is the same as in (A–L). The extreme cases where noise misclassifies the stimulus in a different ISI are shown in red and purple. (N) Sampled PRC corresponding to data in (M), shown plotting sampled PRC against sampled phase. Color-coding corresponds to each of the cases in (A–L).

In order to explain how the ISI variability might affect the PRC calculation, we sketch twelve representative scenarios in which spike jitter due to noise causes misclassification of the phase and/or the PRC value. In these scenarios (shown in Fig. 2A–L), we jittered either the first or the second AP (Fig. 2A–L, black lines) with respect to a perfectly periodic cycle of firing (Fig. 2A–L, grey lines). We divided the sketches into three blocks depending on the phase of the current pulse within the cycle (Fig. 2 A, D, G, J: early phase; B, E, H, K: middle phase; C, F, I, L: late phase). The misclassification of phase and/or PRC value (arrows) becomes clear when comparing them against their deterministic counterparts. The jitter of the spike preceding or following the brief current pulse can lead to a loss of causality and hence to a drastic miscalculation of the PRC. The most serious consequences of the ISI variability due to noise occur in the scenarios illustrated in Fig. 2C and 2J, where the jitter causes the current pulse to fall into a different cycle of firing, resulting in a significant bias at the late and early phases of the PRC, respectively. Specifically, the “Bermuda Triangle” effect present in both model and experiment can be explained by means of the sketch in Fig. 2C: when the pulse arrives at late phases, and the AP jitter results in the pulse falling into the subsequent ISI as compared to the deterministic case, the resulting phase is small according to the new ISI boundaries. Due to causality, it is impossible for a PRC point to fall into the green “Bermuda Triangle” in Fig. 1, since for all points in the triangle the shortening of the ISI would be larger than the actual phase difference of the pulse to the end of the ISI. This explains the observation that phases are sampled less frequently in the late part of the ISI, and thus the PRC values are underestimated and the effect of ISI noise is not averaged out.

**Figure 3:**
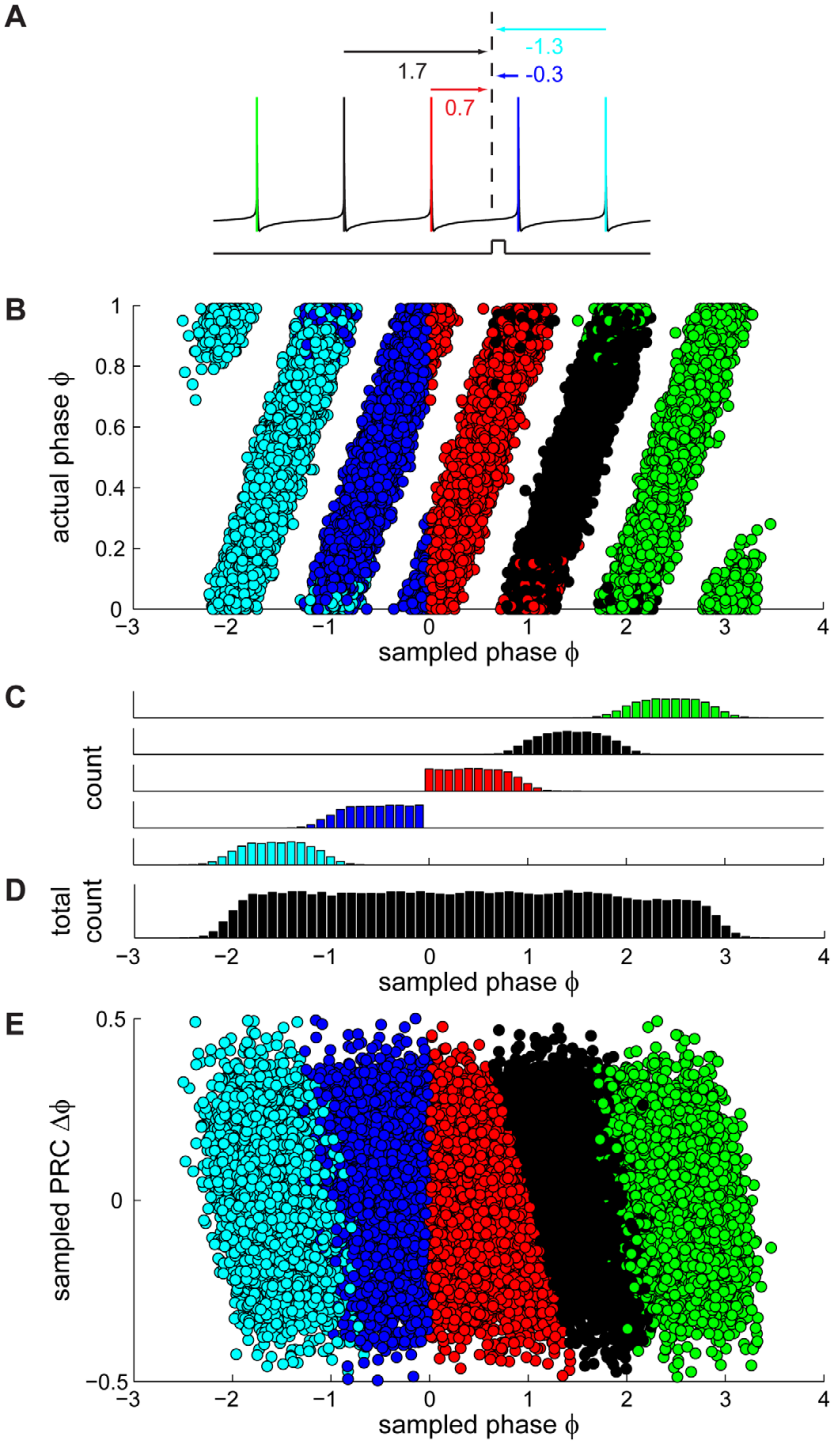
A new approach for generating corrected PRCs. (A) Schematic illustration of a spike train, coded by color: the red spike is immediately preceding the pulse and is the one used as a reference (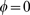) in the traditional method. The remaining colors correspond to the temporal sequence of spiking (green: three spikes before pulse, black: two spikes before pulse, blue: first spike after pulse, cyan: second spike after pulse). In our new approach, all spikes are used as a reference one at a time to calculate the phase of the current pulse (values under arrows) and the corresponding PRC value. The red arrow indicates the phase used to calculate the PRC using the traditional method. (B) Sampled phase vs. actual phase plot as in Fig. 2M with the color code corresponding to the colors used in (A). The two spikes preceding the pulse (red and black circles) contribute to the interval of interest [0,1], whereas in the traditional method only the spike immediately preceding the pulse is used as a reference (red circles). (C) Phase histograms, plotted separately for the different reference spikes in the corresponding color code. (D) Phase histogram of sampled phases using all five spikes as a reference one at a time. (E) Same sampled PRC vs. sampled phase as in Fig. 2N with the same color code as in (A). PRC_2_ and PRC_3_ are calculated by using only the spikes following the pulse as reference spikes, and correspond to the phase intervals [−1,0] and [−2,−1] respectively. The data shown in Fig. 3B–E (as well as 2M,N) assume for the purpose of illustration that the process underlying spiking is perfectly periodic and that the presence of a spike does not reset this underlying process. However, when actually applying our corrected method for calculating the PRC, the phase *is* yoked to the reference spike, i.e. phase is reset at each reference spike.

To visualize the resulting phase and PRC misclassification, we translated each of these twelve sketches onto a corresponding phase plot (Fig. 2M,N). This allows the resulting phase and PRC values of each of the twelve cases to be compared against their deterministic counterparts. More specifically, regularly spaced spike times were defined and jittered independently by noise taken from a Gaussian distribution. The known actual phase without noise was plotted against the sampled phase. The assumption that the process underlying spiking is perfectly periodic and that the presence of a spike does not reset this underlying process is made only for generating the data in Fig. 2M,N (and subsequently Fig. 3B–E), and only for purposes of illustration. In a purely deterministic scenario, the sampled phase is linearly dependent on the actual phase (Fig. 2M, points on the diagonal). This is also the case for occurrences in which the noise has no effect on the phase (e.g. the scenarios in Fig. 2A or 2B; yellow points in Fig. 2M). For any deviations of the sampled phase from the actual phase due to noise, the points are scattered across the plot (Fig. 2M, color coding as in A–L). Based on the same principles, the effect of noise in each of the twelve scenarios on the PRC plot is shown in Fig. 2N (color coding as in A–L).

To summarize, the bias at late phases of the PRC calculated using the traditional method is due to erroneous phase sampling, which results from the substantial ISI variability present in spontaneously firing Purkinje cells, and the loss of causality between the current pulse and the jitter in the times of either of its two surrounding APs.

### Improving the traditional method to obtain PRCs in the presence of noise

Our new method to correct for the bias in the traditional PRC and obtain a homogeneous phase histogram is illustrated in Fig. 3A. The red spike immediately preceding the pulse is the one used as a reference (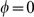) in the traditional method. In our new method, instead of using just the spike immediately preceding the current injection, each spike in the spike train is taken as a reference one at a time and the corresponding phase values (indicated under the arrows in Fig. 3A) are all taken into account (see also Materials and Methods). In this case, the two spikes prior to the stimulation pulse (red and black in Fig. 3A) predominantly contribute to the phase interval [0,1] of the PRC (Fig. 3B, red and black points). The impact of the pulse on the subsequent ISI, the PRC_2_, is then determined by the two spikes following the current pulse (Fig. 3A blue and cyan spikes; also compare [24]) and so on. It is worth emphasizing that even though more than one spike is included in the PRC calculation, the presence of each reference spike resets the phase to zero (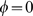). Our method restores periodicity in the spiking jitter as can be seen in Fig. 3B (all points, in analogy to Fig. 2M). By taking only the points according to the traditional calculation of the PRC, a sharp boundary is drawn (Fig. 3B, red) resulting in an inhomogeneous distribution of sampled phases (Fig. 3C, red). In contrast, by including the second spike prior to the pulse, spike jitter effects are averaged out (Fig. 3D). The bias at the late phases of the PRC plot observed when taking points according to the traditional calculation of the PRC (Fig. 3E, red) is thereby eliminated (Fig. 3E, all points), as is the bias in the cPRC (not shown).

In order to validate our new method, we applied it to neuronal models for which the PRC can be calculated analytically (from the adjoint [27]). PRCs of the Morris-Lecar model (parameters from [28]), in the presence and absence of noise, were compared with the analytically derived PRC (Fig. S1A). The PRCs calculated using both the traditional and our corrected method overlap perfectly (except near 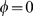 and 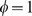, due to the finite time step and finite amplitude of the current pulse in the simulations), and match the analytically derived PRC. In the presence of noise, the PRC calculated by the traditional method is biased at late phases, as described above. Our new method eliminates most of this bias.

However, it has been shown that noise can directly affect the dynamics of neurons underlying the PRC, leading to changes in the PRC which are not due to measurement errors (e.g. in the Morris-Lecar model [29]). We therefore used an additional model, the non-leaky integrate-and-fire model, in which noise-dependent changes of dynamics can be excluded. When noise was introduced in this model, the traditional method resulted in a biased PRC, as compared to the analytically derived PRC and the PRC in the absence of noise. Again, our corrected method removed most of this bias (Fig. S1B). The same analysis was repeated in a leaky integrate-and-fire model. This shows that the “Bermuda Triangle” and its consequences on the PRC are the result of the traditional calculation of PRCs, separate from the effect of noise on the dynamics of the system (Fig. S1C).

**Figure 4:**
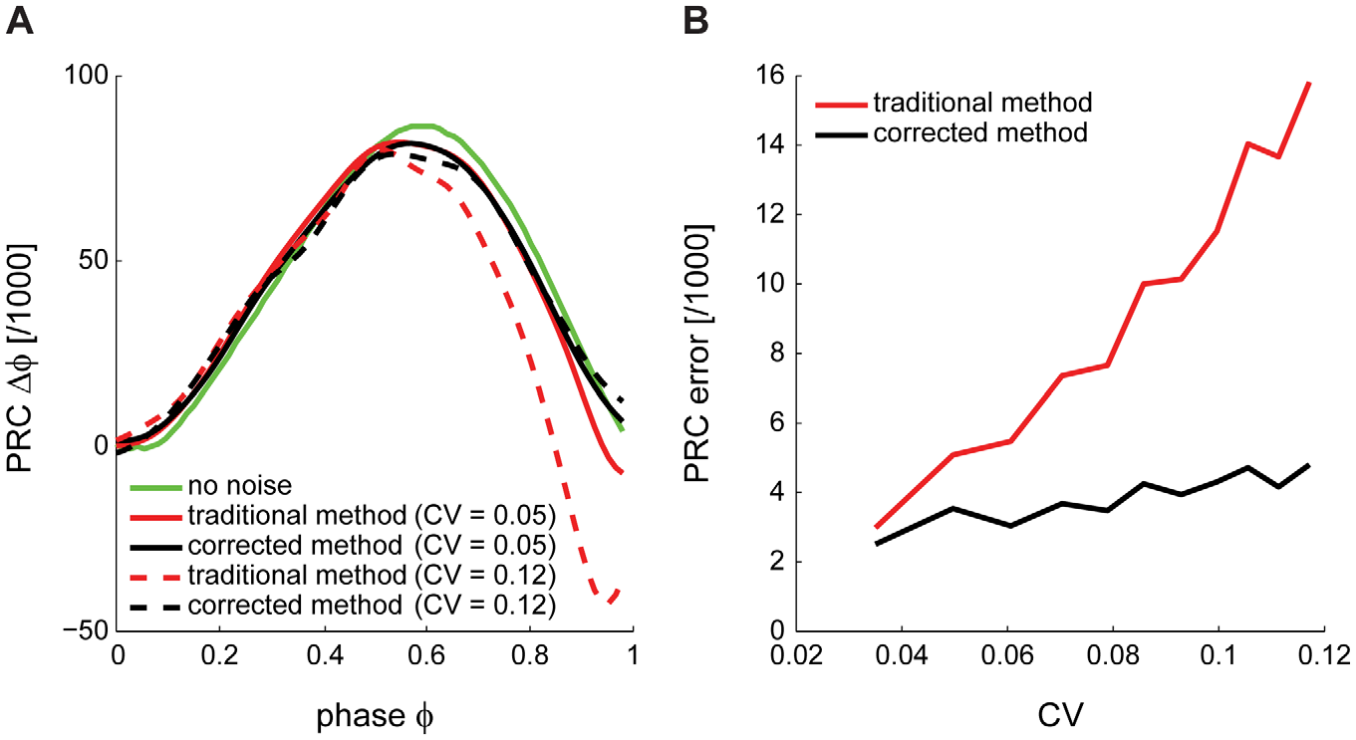
Validation of the corrected method for obtaining PRCs. (A) PRCs of a model Purkinje cell, calculated using the corrected method in the stochastic model (black), in the deterministic model (green) and calculated using the traditional method in the stochastic model (red). Traces are shown at two different noise levels (increased CV indicated by dashed red and dashed black lines respectively). (B) PRC error, expressed as the integral of the differences between PRCs and their deterministic counterparts. The corrected method performs better than the traditional method in all cases, particularly at high CV.

Next, using the Purkinje cell model [26], we compare the result of our method (Fig. 4A, black) to the PRC obtained with the traditional method (Fig. 4A, red) and the deterministic PRC without noise (Fig. 4A, green). When the noise is increased, reflected by an increased coefficient of variation (CV) of ISIs, the traditional PRC deviates from the deterministic one and the bias becomes more pronounced (Fig. 4A, dashed red line). In contrast, our corrected method performs as well as with low CV (Fig. 4A, dashed black line). The strong bias at late phases is eliminated. In order to evaluate the performance of our method in comparison to the traditional method, we calculated the integral of the differences between PRCs and their deterministic counterparts (PRC error; Fig. 4B). As the CV increases, the PRC error shows larger increases using the traditional (Fig. 4B, red line) compared to our corrected method (Fig. 4B, black line).

In conclusion, the “Bermuda Triangle“ present in PRCs is due to shortcomings of the traditional method for calculating PRCs. The bias can for the most part be compensated for by taking the two spikes preceding the pulse as a reference, one at a time, instead of just the spike immediately preceding the pulse as in the traditional method.

### A frequency-dependent switch in Purkinje cell dynamics

Spontaneous firing frequencies of Purkinje cells range from 10–180 Hz both *in vitro* [1], [2], [4], [5] and *in vivo* [3], [30]. To test how the dynamics of Purkinje cells change according to the firing frequency, we recorded from cells firing spontaneously at low (15–40 Hz, n = 10) and high (55–180 Hz, n = 6) rates and calculated their PRCs using our corrected method.

**Figure 5:**
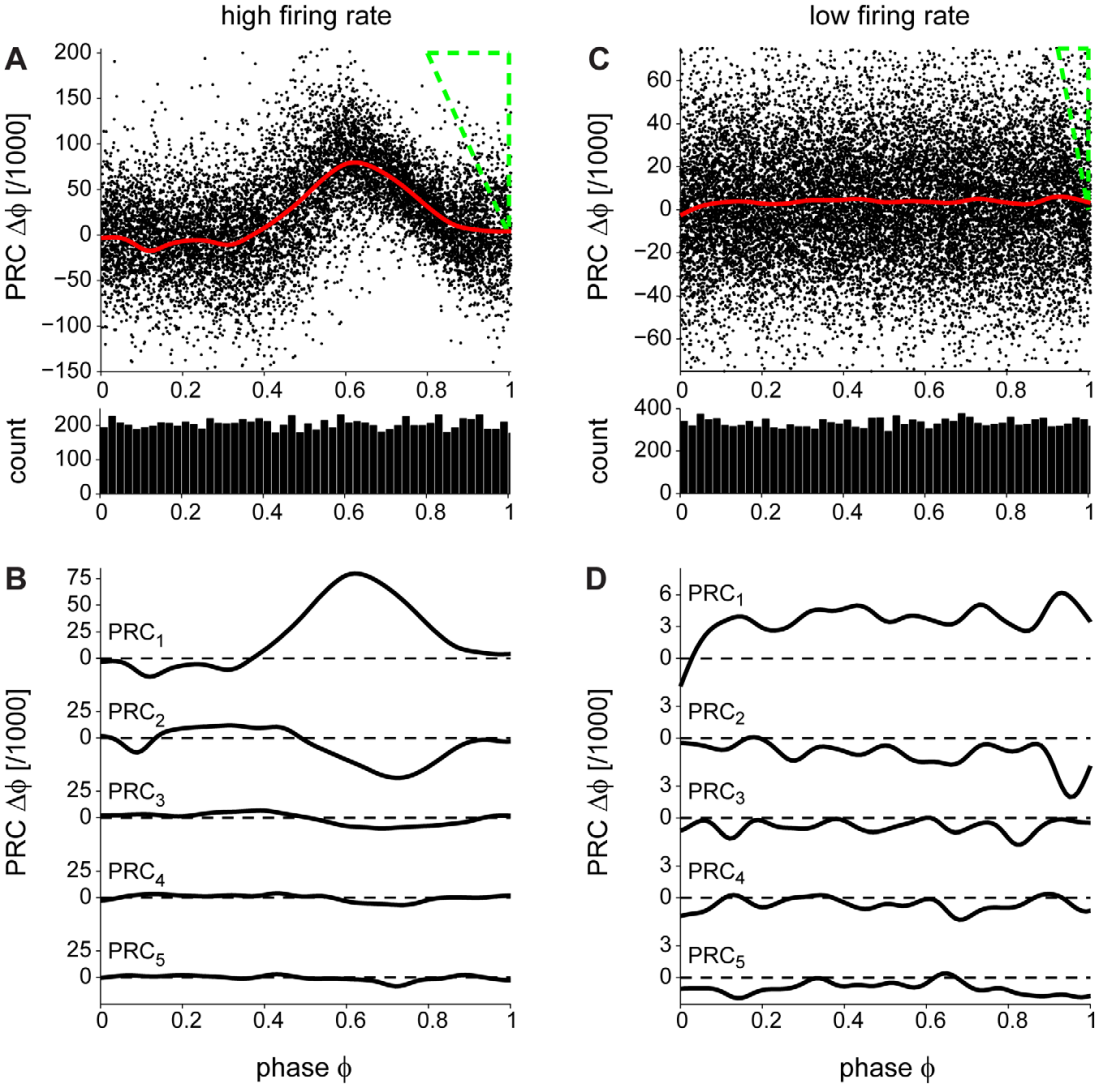
Two types of PRCs depending on the Purkinje cell firing rate. (A) PRC of a rapidly firing cell. Using the corrected method the “Bermuda triangle” (shown in green dashed lines) and the bias at the late phases are eliminated. The brief current pulses cause an ISI shortening as reflected by the positive PRC values. The panel below shows the phase histogram, which is homogeneous, suggesting an equally sampled ISI. (B) The effect of the brief current pulse is persistent in the subsequent ISIs (PRC_2–5_) and dies out after four ISIs. PRC_2–5_ are negative suggesting ISI lengthening. (C) PRC of a slowly firing cell. The PRC is square/flat; the brief current pulse causes the same effect independent of its position within the ISI. Again, the phase histogram, below, is homogeneous revealing an equally sampled ISI. (D) Similarly to (B), the brief current pulse lengthens subsequent ISIs: PRC_2–5_ are negative.

A representative corrected PRC is shown in Fig. 5A (the same example of a rapidly firing (180 Hz) Purkinje neuron as in Fig. 1B). The PRC is positive, indicating that the brief current pulse causes an advance of the following spike (shortening of the ISI relative to the mean) with maximum displacement when the pulse arrives near the middle of the ISI. It is worth noting that the phase histogram is homogeneous (Fig. 5A, lower panel), suggesting that, with the corrected method, the ISI is equally sampled throughout. In order to study the effects of the brief pulse on the subsequent intervals we plotted the PRC_2–5_ (Fig. 5B; see Materials and Methods). PRC_2_ is negative, suggesting that the subsequent ISI is lengthened relative to the mean. A PRC_2_ with opposite sign to the PRC has been previously reported [24] and it is believed to be due to a compensatory effect on the current ISI length. Indeed, as seen from PRC_2–5_, these curves are negative and the effect dies out after about 4 ISIs.

In comparison, an example of a PRC of a slowly firing (30 Hz) Purkinje neuron is shown in Fig. 5C. The brief current pulse causes the same positive displacement of the following spike independently of its position within the ISI, resulting in a square PRC. The phase histogram is homogeneous, indicating that there is an equal probability for the pulse to arrive at each phase within the ISI (Fig. 5C, lower panel). In order to study the effects of the brief pulse on the subsequent intervals we calculated the PRC_2–5_ (Fig. 5D; see Materials and Methods). They were negative, similar to those of cells firing at a high rate, but exhibited larger fluctuations. It is interesting to note that the PRC phase advances occur at a different scale in the slowly and rapidly firing Purkinje cells. However, when converted back into time units, the PRC values are of the same order of magnitude in both cases (see below).

**Figure 6:**
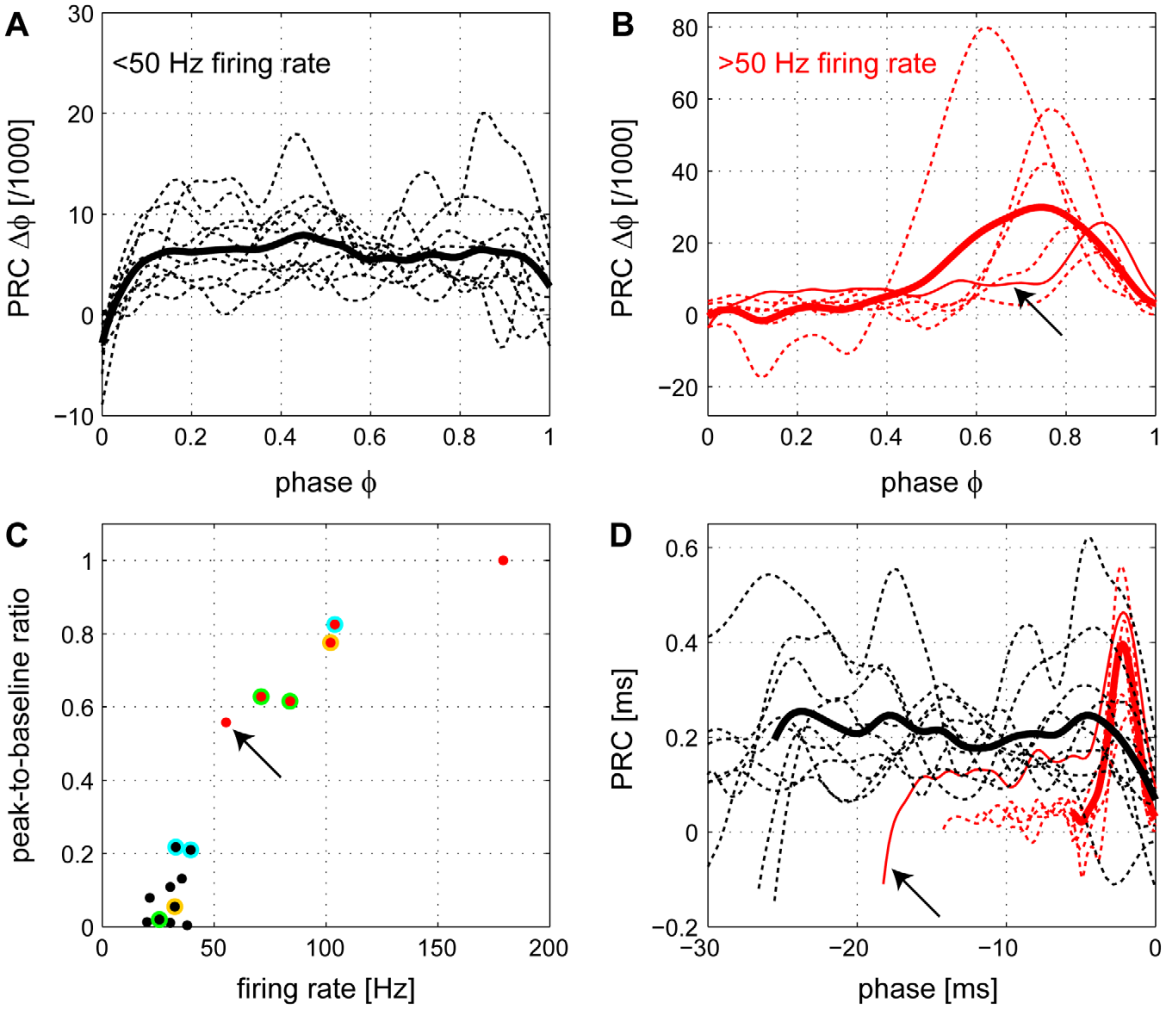
A frequency-dependent switch in Purkinje cell dynamics. (A) Individual PRCs (thin dashed lines) and the average PRC (thick line) of slowly firing cells (frequency<50 Hz, n = 10, black). The average PRC is independent of the phase of the brief current pulse within the ISI. (B) Individual PRCs (thin lines) and the average PRC (thick line) of rapidly firing cells (frequency>50 Hz, n = 6, red). The PRC is phase-dependent with a peak at late phases. The thin solid line is the PRC of the cell with the lowest firing frequency (55 Hz) in this group (arrows in (B–D) and shows both a peak at late phases and a plateau at early phases. (C) Peak-to-baseline ratio (see Materials and Methods) plotted against firing frequency: as the frequency increases the PRC switches from phase-independent to phase-dependent. Points shown in two colors correspond to PRCs from the same cell (see Figure 7 and text for more details). (D) PRCs of the two groups of cells (black: low firing rate, same data as in (A); red: high firing rate, same data as in (B) plotted on the same axes with both the phase and the phase shift of the PRC in units of time, and phases aligned with respect to the second AP in the ISI. The peaks seen at high firing rates coincide at a time of approximately –3 ms from the second spike.

The PRCs of Purkinje cells exhibiting slow (15–40 Hz; n = 10) and rapid (55–180 Hz; n = 6) spontaneous firing were calculated using our corrected method. The PRCs switched from square (phase-independent) for lower frequencies (Fig. 6A) to phase-dependent for higher frequencies (Fig. 6B). The switch occurred at a frequency of approximately 50 Hz. The average PRC of all neurons firing at low rates (Fig. 6A, thick line) is phase-independent. To our knowledge, such a square PRC has not been previously reported. A square PRC can only be obtained if the cells act as perfect non-leaky integrators. In contrast, the average PRC of all Purkinje cells firing at high rates (Fig. 6B, thick line) exhibited a sharp peak. It is useful to compare these average PRCs (Fig. S2, thick black and red lines) with the biased ones obtained with the traditional method (Fig. S2, thick green lines). To quantitatively assess the switch in dynamics we plotted the peak-to-baseline ratio of the PRCs in relation to the firing rate (Fig. 6C; see Materials and Methods). This quantity essentially compares the extreme value in the first half of the PRC with the extreme value in the second half. The switch at a firing rate of approximately 50 Hz can be seen clearly in this representation. The switch becomes particularly apparent when both the phase and the phase shift of the PRC are plotted in units of time, and phases are aligned with respect to the second AP in the ISI (Fig. 6D). Then, the peaks of the PRCs measured at high firing rates coincide (red), indicating that an input signal causes an effect only in a 3 ms window prior to the output spike irrespectively of the precise firing rate of the cells in that group. This peak in the PRC is shown to give way to a larger phase-independent plateau (black) at low firing rates, in which incoming signals will affect the spiking of the cell regardless of the time at which they arrive. A transitory PRC (thin solid red lines in Fig. 6B and Fig. 6D, indicated by arrows) showing both a plateau at early phases and a peak at late phases was observed in a cell with intermediate firing frequency (55 Hz). To summarize, the PRCs of Purkinje cells largely depend on the intrinsic firing frequency of the cells: they are phase-independent at low firing rates (15–40 Hz) and phase-dependent at high frequencies (55–180 Hz).

**Figure 7:**
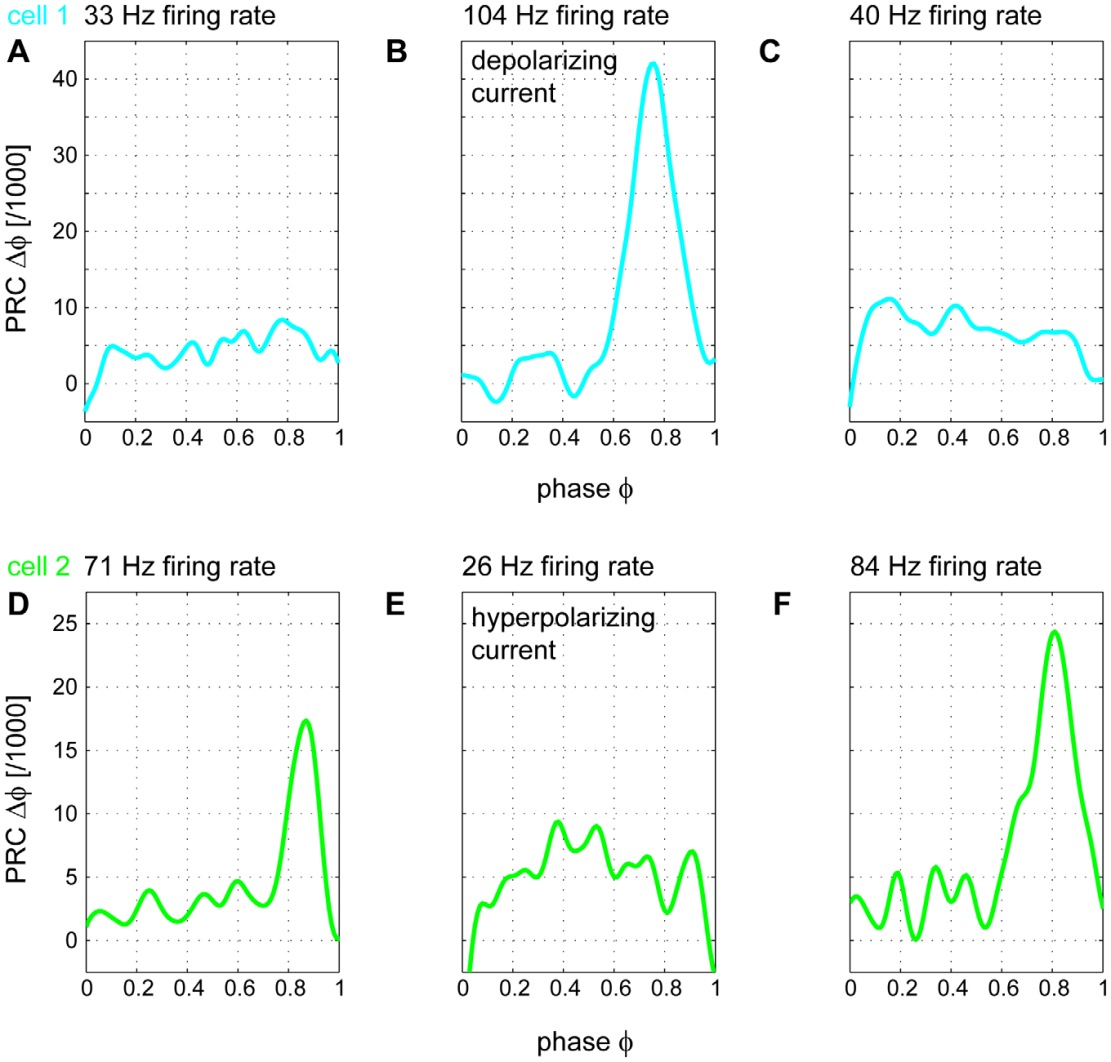
The switch in PRC shape can occur within the same cell. (A) A slowly firing cell (33 Hz) displaying a phase-independent PRC. (B) Injection of depolarizing current into the cell in (A) increases its firing rate (104 Hz) and the PRC switches to being phase-dependent. (C) When the depolarizing current is removed, the cell relaxes back to its intrinsic spontaneous firing rate (40 Hz) and its PRC switches back to being phase-independent. (D) A rapidly firing cell (71 Hz) displaying a phase-dependent PRC. (E) When hyperpolarizing current is injected into the cell to decrease its firing rate (26 Hz), the PRC switches to being phase-independent. (F) Removal of the hyperpolarizing current returns the cell to its intrinsic spontaneous firing rate (84 Hz) and its PRC switches back to being phase-dependent.

The firing rate of a Purkinje cell changes depending on modulation of its inputs [31]–[35]. For example, during locomotion in cats the firing frequencies of Purkinje cells can increase from an average of about 40 Hz [34] to more than 100 Hz [35]. To test whether the switch in Purkinje cell dynamics can occur in the same cell, we recorded Purkinje cell PRCs while modulating their firing frequencies using injected current (n = 3; Fig. 6C, points labeled with two colors). We first recorded at the spontaneous firing frequency, and if the spontaneous frequency was low, we next increased the firing rate by injecting a positive constant current. Alternatively a negative constant current was injected if the spontaneous frequency was high. The PRCs for both fast and slow states were calculated (Fig. 7, color coding as in Fig. 6C). When Purkinje cell spiking was changed from slow (33 Hz) to fast (104 Hz), the originally square PRC (Fig. 7A), exhibited a sharp peak (Fig. 7B). This change in the PRC was reversible, as when the neuron was allowed to relax back to its intrinsic firing rate (40 Hz) the PRC returned to a square shape (Fig. 7C). Conversely, another neuron initially firing at a high rate (71 Hz) exhibited a peaked PRC (Fig. 7D), which was switched to a square shape by reducing its firing rate to 26 Hz via injection of hyperpolarizing current (Fig. 7E). When the neuron was then allowed to fire at its intrinsic firing rate (84 Hz) the sharp peak in the PRC reappeared (Fig. 7F). Therefore, the switch in Purkinje cell dynamics reflected in the switch of the PRC can also occur in the same cell.

## Discussion

We have shown that the traditional method for calculating PRCs results in a bias, particularly in neurons exhibiting high ISI variability. We developed a corrected method for calculating PRCs which removes most of this bias. Our method can be directly applied to noisy experimental data. We used this corrected approach to measure for the first time the PRCs of Purkinje cells at various firing rates. At high firing rates, Purkinje cell PRCs were phase-dependent; however, a phase-independent PRC was observed at lower firing rates. This suggests that Purkinje cells can behave as perfect integrators at low firing rates, which has important consequences for our view of the integrative properties of these neurons.

### A new approach for determining PRCs

We have determined Purkinje cell PRCs by injecting brief current pulses and measuring the phase change in the subsequent neuronal spiking. Since at the typical spontaneous firing rates of Purkinje cells these phase changes were small compared to the spike jitter during spontaneous spiking [1], many trials were required. This revealed a general bias of the traditional method at late phases of the PRC in the presence of noise (Fig. 1). We characterized the effect in a model with and without noise, and showed that the bias is related to inhomogeneous phase histograms caused by interspike interval jitter (Fig. 1 and Fig. 3). To correct for this, we developed a new method, which recovers periodicity in the spike jitter due to noise (Fig. 3). We showed that this method homogenizes the phase sampling in the experimental data and removes most of the bias observed in the PRCs calculated using the traditional method (Fig. 4A). Our corrected approach can be directly applied to existing experimental data in order to measure PRCs under low signal-to-noise conditions. It should be applicable to a wide range of cell types, as neuronal noise and the resulting ISI variability are not restricted to Purkinje cells [36].

The use of indirect methods to obtain PRCs, for example from the spike triggered average [23] or the poststimulus time histogram (PSTH) [24] are possible alternatives to the traditional method. Here we have applied a correction to the traditional method, which resulted in reliable PRC measurements in Purkinje cells. Further alternative methods for calculating PRCs exist. For example, dynamic clamp was previously used to study hippocampal spike-timing-dependent plasticity in relation to PRCs [37]. In this special case, underlying subthreshold oscillations provide phase locking. Such a method is only applicable if phase information is accessible to the experimenter, independent of spiking. PRCs can also be calculated using Bayesian statistics [25], or by injecting trains of rectangular current pulses [38] and noisy inputs [11]. These methods result in periodic PRCs, but only because periodicity is imposed as part of the optimization (fitting) techniques employed. In conclusion, our method can be applied to noisy experimental data to calculate PRCs while avoiding possible bias or overfitting problems present in some of the currently available methods. A wide, comparative study will be required in the future to find out which methods for calculating the PRC yield the best results under different conditions.

### Purkinje cell dynamics depend on firing rate

Purkinje cells fire spontaneously and modulate their firing in response to synaptic input. The spontaneous firing rate of Purkinje cells varies from 10 to 180 Hz, but firing frequency can also be increased by the ∼150,000 parallel fiber synaptic inputs [39] or decreased by molecular layer interneurons during the execution of motor tasks such as smooth-pursuit eye movements [40], maintenance of posture [41] and locomotion [35], [42]. For example, the rate of Purkinje cell firing can exhibit a consistent temporal relationship with wrist movement [31] or be monotonically related to eye velocity during smooth-pursuit eye movements [40].

How is the integration of single inputs affected by the firing rate of the Purkinje cell? We have addressed this question by measuring the PRC at different firing rates. Using our new approach, we determine experimentally the PRCs of cerebellar Purkinje cells and show that their shape changes significantly depending on the firing rate (compare Fig. 5A and Fig. 5C). At high firing frequencies (>50 Hz) Purkinje cell PRCs are monophasic (Fig. 6B). However, at low firing rates (<50 Hz), Purkinje cell PRCs become phase-independent (Fig. 6A). To the best of our knowledge, this is the first study to report a phase-independent PRC in a mammalian neuron.

It was previously reported in a spike-frequency adaptation model of cortical neurons that an increase in firing frequency causes a shift of the PRC peak from rightward skew to the centre with a decrease in amplitude [24], implying that the integrative properties of this model neuron change depending on the firing rate. Specifically, it was suggested that the model cell acts like a coincidence detector at low firing rates and more of an integrator at higher firing rates [24]. Purkinje cells appear to show the opposite behaviour, acting as perfect integrators at low firing rates.

### Functional implications

The shape of the PRC is thought to be linked to the type of excitability of the neuron. Neurons with type I excitability, whose f-I curves are continuous, are thought to display purely positive PRCs while neurons with type II excitability, characterized by a discontinuity in the f-I curve at the onset of firing, exhibit biphasic PRCs [11], [13], [14]. While biphasic PRCs intuitively result in resonator behavior, neurons with purely positive PRCs act as integrators of synaptic input [11], [13], [14], [43]. Although Purkinje cells exhibit type II excitability [2], [44], [45], their PRCs are positive at all firing rates, implying that they are integrators rather than resonators. These findings suggest that the type of excitability of a neuron is not strictly correlated with the PRC shape. Similarly, Tateno and Robinson [15] showed that low-threshold spiking, fast spiking and non-pyramidal regular spiking interneurons can exhibit both purely positive and biphasic PRCs which do not always strictly correspond to the type of excitability of the neuron.

The shape of the PRC has functional implications for the integration of synaptic inputs. At high firing rates, Purkinje cells are most sensitive to inputs during the last 3 ms of their firing cycle (Fig. 6D), imposing a strict relationship between the timing of the input and the timing of spike output, with direct consequences for network dynamics. It has been shown theoretically that oscillators which are described by type I PRCs and are coupled by excitatory synapses tend not to synchronize [16]. However, the opposite is true for inhibitory coupling between oscillators [16], [46], such as coupled Purkinje cells. Indeed, theoretical and experimental evidence indicates that Purkinje cells tend to synchronize via inhibitory inputs [4], [6], [7].

As the firing rate of Purkinje cells decreases, and the levels of synaptic and intrinsic conductances and currents are modified, the PRC switches from monophasic to phase-independent (Fig. 6C). The phase-independent PRCs at low firing rates suggest that Purkinje cells integrate their synaptic inputs independently of their timing within the interspike interval (Fig. 6A). Our results therefore support the idea that at low firing rates, Purkinje cells cannot read out the timing of their inputs, which would exclude the use of a temporal code. Instead, in this regime they are well suited for rate coding.

What are the biophysical mechanisms responsible for the switch in PRC behaviour at different firing rates? To generate an entirely flat PRC would require a neuron to effectively completely compensate for its leak conductance. This is illustrated by the example of the PRC of a simple leaky integrate-and-fire neuron in which the leak conductance was eliminated (Fig. S1B and C). However, this absence of leak is unlikely to occur in real Purkinje cells, and the biophysical implementation remains unknown. PRCs qualitatively similar to those observed in our experiments at high firing rates can be generated by the Purkinje cell model of Khaliq and colleagues [26] (Fig. S3A). However, when the firing rate is lowered in the model, no qualitative switch in the shape of the PRC can be observed. A hint to the mechanisms underlying the switch in the experiment is provided by using the model of Akemann and Knöpfel [47] (a further development of the Khaliq et al. model): at low firing rates a ‘shoulder’ appears in the early phases of the PRC (Fig. S3B). However, none of these models fully capture the experimentally determined switch in Purkinje cells, perhaps reflecting the fact that both of these models represent dissociated Purkinje cells. Thus, our experimental results could aid the refinement of existing models in order to capture the full dynamic behaviour of Purkinje cells.

In conclusion, our experimental findings indicate that Purkinje cells display different dynamic behavior depending on their firing rate. At high firing rates these neurons act as coincidence detectors of synaptic inputs, with maximal sensitivity at the late phases of the interspike interval. In contrast, at low firing rates Purkinje cells are not suited for precise coincidence detection, but instead appear to perfectly integrate their inputs independently of their position within the interspike interval. Thus, at high firing rates Purkinje cells can transmit information via a temporal code whereas at low firing rates they are well-suited for rate coding.

## Materials and Methods

### Ethics statement

All procedures were approved by the U.K. Home Office.

### Acute slice preparation and electrophysiological recordings

Twelve- to fifteen-day-old *L7-tau-gfp* mice [48] were anaesthetised using isoflurane, decapitated and their brains were transferred to ice-cold low Ca^2+^ artificial cerebrospinal fluid (ACSF) containing (in mM): 125 NaCl, 26 NaHCO_3_, 25 glucose, 2.5 KCl, 26 NaH_2_PO_4_, 0.5 CaCl_2_ and 3 MgCl_2_, saturated with carbogen (95% oxygen and 5% carbon dioxide gas). 230-µm-thick sagittal brain slices from the cerebellar vermis were cut on a VT1200S microtome (Leica Microsystems) and were transferred to normal ACSF containing the following (in mM): 125 NaCl, 26 NaHCO_3_, 25 glucose, 2.5 KCl, 26 NaH_2_PO_4_, 2 CaCl_2_ and 1 MgCl_2_, again bubbled with carbogen. The slices were incubated for 30–40 minutes at 37°C and were then allowed to cool to room temperature. Thick-walled, filamented, borosilicate glass electrodes (Harvard Apparatus Ltd.) were pulled to a tip resistance of 4–5 MΩ (PC-10 microelectrode puller, Narishige). Cells were visually identified with the aid of an upright infrared differential interference contrast (IR-DIC) microscope (Axioskop, Carl Zeiss) and a video camera (C2400-07, Hamamatsu). Purkinje cell somatic whole-cell patch-clamp recordings were obtained using an internal solution containing the following (in mM): 130 methanesulfonic acid, 10 HEPES, 7 KCl, 0.05 EGTA, 2 Na_2_ATP, 2 MgATP, 0.5 Na_2_GTP and 0.4% biocytin, pH-adjusted to 7.3 with KOH. All recordings were performed at 34.5±1°C in the presence of carbogen-bubbled ACSF supplemented with GABA_A_ receptor blocker SR95331 (10 µM). Recordings were made with an Axoclamp 2B amplifier (Axon Instruments) and were filtered at 3 kHz and sampled at 50 kHz using an ITC-18 DAC board (Instrutech) and Axograph 4.9 (Axon Instruments). Series resistance and pipette capacitance were carefully monitored and compensated throughout the experiment. Methanesulfonic acid was obtained from Fluka, and other chemicals from Sigma-Aldrich and BDH Chemicals.

### Phase response curves

Data were analyzed with MATLAB (The MathWorks). To determine how spike timing during spontaneous firing is shifted by a brief perturbation, we injected rectangular current pulses of 0.5 ms duration and 50 pA amplitude, after a baseline of 150 ms (50 ms) of spontaneous firing in subsequent trials of 350 ms (100 ms) for a slowly (rapidly) firing cell. A control PRC (cPRC) was calculated using the unperturbed part of the voltage traces and assuming a current pulse injection (0 pA amplitude) after 50 ms (25 ms) of spontaneous firing in subsequent trials of 350 ms (100 ms) for a slowly (rapidly) firing cell. The cPRC should be zero throughout all phases.

The dynamics of a neuronal oscillator can be reduced to a single variable: the phase 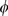. 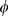 is calculated by dividing the time from the previous spike by the period 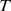 of the oscillation; it increases linearly from 0 to 1 between two spikes. Depending on the phase 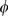 of the stimulus, a change in phase, 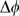, of subsequent spiking will occur.

Traditional method: A brief current pulse is injected at a random time. The spikes before and after it are identified. 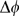 is calculated by the difference between the unperturbed 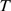 and the perturbed 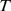 [8]–[12]. When the unperturbed 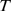 is defined as the mean ISI (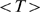), a point 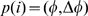 on the PRC plot becomes:
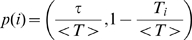
(1)where 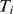 denotes the ISI which contains the brief current pulse and 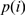 is the PRC point calculated in reference to the spike just prior to the stimulus. 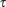 is the time between the pulse and the preceding spike 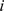. The resulting curve is a plot of 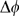 against 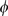. The curve is positive (negative) when the injected current advances (delays) the next spike. In the experimental and model (with noise) PRCs, we refer to raw data as the estimated measurements (‘points’) on the PRC plot. A moving average was calculated with a Gaussian kernel over the raw data.

Corrected method: A major problem with the traditional method is the loss of periodicity of the sampling reference (Fig. 3B), which results in an inhomogeneous sampling of phases in the presence of spike jittering. In order to restore periodicity, points unaffected by the stimulation pulse can be added to the ensemble of PRC points, which allows the spiking jitter to average out properly. These points can be obtained from the same data by adding PRC values when the preceding ISI is taken into account:
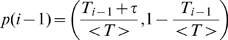
(2)When 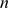 preceding and 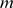 subsequent ISIs are taken into account as in:
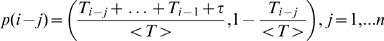
(3)and
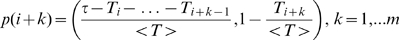
(4)periodicity in the spiking jitter is restored, phases are sampled homogeneously and the cPRC becomes flat. In the resulting plot, the phase interval ranges from 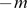 to 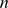 and the PRC component affecting directly the interval 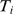 corresponds to all points in the phase interval [0,1], termed PRC_1_. Successive PRC_2–5_, correspond to phase intervals [−1,0], [−2,−1], [−3,−2] and [−4,−3], respectively and indicate how 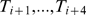 are affected by the pulse.

*Peak-to-baseline ratio:* In order to distinguish the phase-independent PRCs from the phase-dependent ones, PRCs were classified according to the peak- to-baseline ratio. Inspired by Tateno and Robinson [15], local extrema at the two halves of the PRC (i.e. for 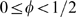 and 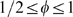) were calculated and were denoted as early (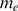) and late (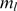) respectively. The peak-to-baseline ratio is then defined as:
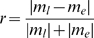



### Simulations

Simulations were performed in NEURON [49] using a model of Purkinje cells consisting of a single compartment [26], [47]. The model includes seven voltage-gated conductances (a resurgent Na^+^ current, fast and slow K^+^ currents, P-type Ca^2+^ current, Ca^2+^-activated K^+^ current and the hyperpolarization-activated current I_h_) and one voltage-independent conductance (I_leak_), based on voltage clamp measurements from Purkinje cells [26]. The membrane surface area of the neuron was modified (×13) to reproduce input resistance values close to those observed in Purkinje cells (80 MΩ). In order to mimic the noise observed in Purkinje cells, noisy current input drawn from a normal distribution with 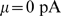 (mean) and 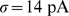 (standard deviation) was injected at each time step of the simulation (every 25 µs) into the soma. The noise injection resulted in a coefficient of variation of ISIs of 0.05, which is comparable to the values measured in real Purkinje cells in the experiments presented here (see also [1]). Current pulses of 0.5 ms duration and 250 pA amplitude were injected after 2500 ms, at a time at which spike jitter had randomized spiking phase. Data shown is taken from more than 15000 trials.

Additional neuron models were used in the supplementary parts of the manuscript. For Fig. S1, the Morris-Lecar model was directly implemented using parameters from [28]. The adjoint was calculated using XPPAUT [27] and the PRCs with noise were integrated in MATLAB (The MathWorks). The parameters for the leaky integrate-and-fire model were: a membrane time constant of 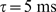, a reset potential of 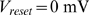, a threshold potential of 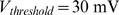, a membrane resistance of 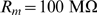, and a steady driving current of 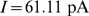 (to result in 50 Hz firing) and was simulated at time steps of 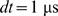. For the non-leaky integrate-and-fire model the time constant 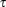 was set to infinity and 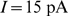, otherwise the same parameters were used. An alternative model for Purkinje cell firing was used for Fig. S3 which also includes a resurgent Na^+^-current and modified voltage-gated K^+^-conductances [47]. In this model, current pulses of 0.5 ms duration were injected at amplitudes of 10 pA in the low firing rate (33 Hz) case and 60 pA in the high firing rate (111 Hz) case. Simulation results were analysed in the same way as the experimental data.

## Supporting Information

Figure S1 Validation of the corrected method. (A) Comparison between the traditional method (red line) and the corrected method (black line) to obtain PRCs and their numerical (green line) and analytical (blue dashed line) no-noise pendants (using the example of the Morris-Lecar model for which the analytical PRC can be calculated by the adjoint method). Curves have been rescaled to their maxima to aid comparison. (B) and (C) Same procedure for a non-leaky and a leaky (5 ms time constant) integrate-and-fire (I&F) model. In all cases the corrected method performs better than the traditional method. The use of the corrected method is particularly important in (B) which corresponds best to the case observed in the experimental data from Purkinje cells at low firing rates. Note that here we find phase-independent and phase-dependent PRCs in a simple I&F neuron. A square PRC can be obtained by eliminating the leak of the I&F neuron, compare (B) and (C). Purkinje cells therefore act as perfect non-leaky integrators at low frequencies (compare Fig. 6A). A phase-dependent PRC can be obtained by adding a leak to the I&F neuron (right). This suggests that Purkinje cells act like leaky integrators at high frequencies (compare Fig. 6B).
(0.52 MB TIF)

Figure S2 Comparison of the corrected and traditional methods for obtaining PRCs. (A) and (B) PRCs of individual Purkinje cells firing at low rates (A, black) and high rates (B, red) and population averages (thick lines), exactly as in Figure 6AB of the main manuscript. (C) and (D) PRCs of the same cells as in (A) and (B) but obtained using the traditional method. The population averages obtained with the traditional method (thick green lines) are qualitatively different from the population averages obtained using the corrected method (thick black and red lines). The bias is such that the conclusions obtained in this study would not have been possible without developing the new method.
(0.32 MB TIF)

Figure S3 PRCs in different model neurons. (A) PRCs obtained with the model of Khaliq et al. [26] at a low firing rate (left) and a high firing rate (right) both exhibit monophasic behavior as seen in the experiment at high firing rates. (B) Frequency dependence of PRCs in an alternative Purkinje cell model from Akemann and Knöpfel [47]. A hint of a ‘shoulder’ at low firing rates indicates the trend of a frequency dependent switch in the model. However, the PRCs at low firing rates (left) are still not flat.
(0.21 MB TIF)
