# Supplementary figures and images for: A New Approach for Determining Phase Response Curves Reveals that Purkinje Cells Can Act as Perfect Integrators
Source: PLoS Comput Biol. 2010 Apr 29;6(4):e1000768. doi: 10.1371/journal.pcbi.1000768 (PMC2861707; doi:10.1371/journal.pcbi.1000768)

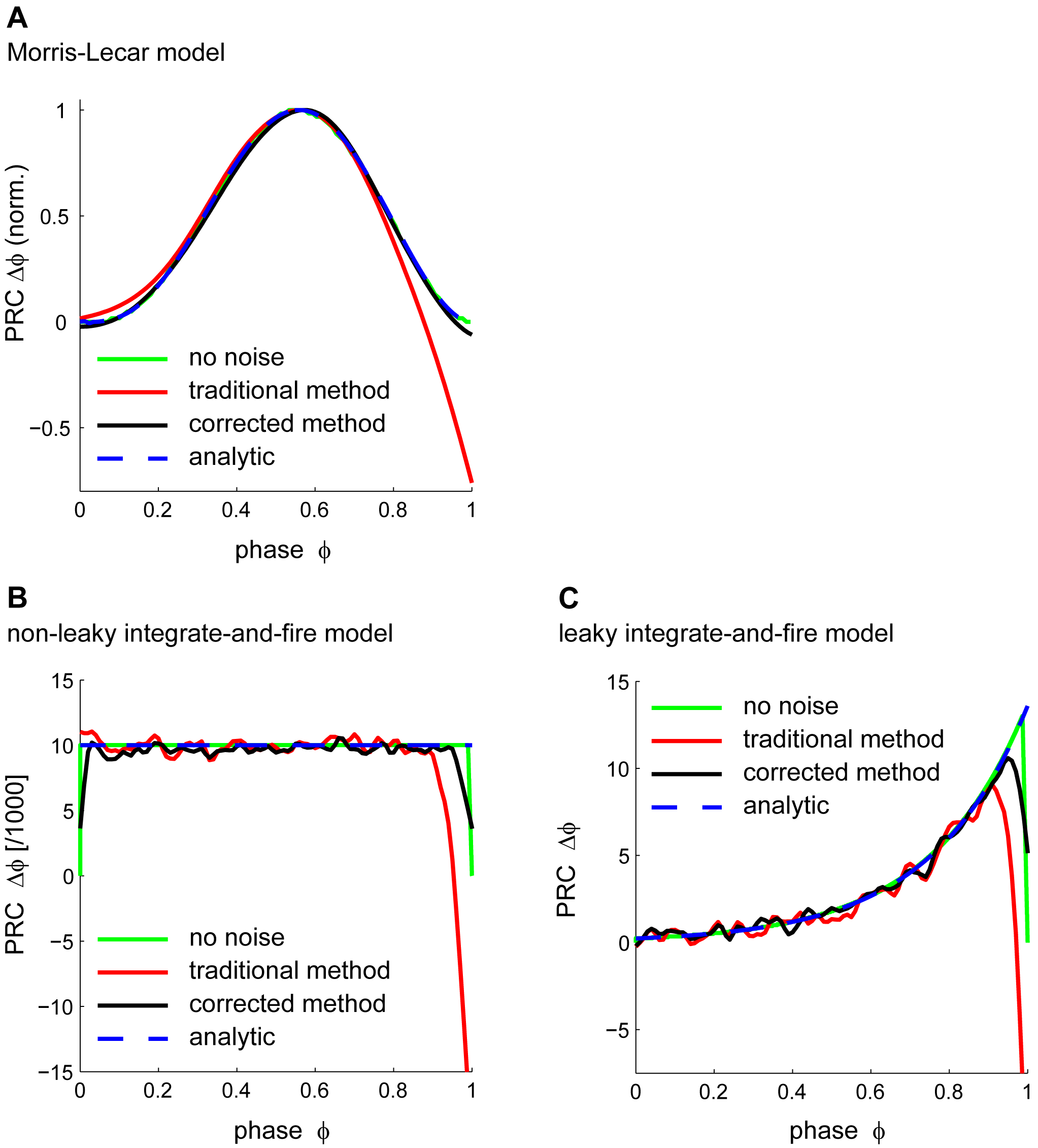

Supplement: Figure S1 — Validation of the corrected method. (A) Comparison between the traditional method (red line) and the corrected method (black line) to obtain PRCs and their numerical (green line) and analytical (blue dashed line) no-noise pendants (using the example of the Morris-Lecar model for which the analytical PRC can be calculated by the adjoint method). Curves have been rescaled to their maxima to aid comparison. (B) and (C) Same procedure for a non-leaky and a leaky (5 ms time constant) integrate-and-fire (I&F) model. In all cases the corrected method performs better than the traditional method. The use of the corrected method is particularly important in (B) which corresponds best to the case observed in the experimental data from Purkinje cells at low firing rates. Note that here we find phase-independent and phase-dependent PRCs in a simple I&F neuron. A square PRC can be obtained by eliminating the leak of the I&F neuron, compare (B) and (C). Purkinje cells therefore act as perfect non-leaky integrators at low frequencies (compare Fig. 6A). A phase-dependent PRC can be obtained by adding a leak to the I&F neuron (right). This suggests that Purkinje cells act like leaky integrators at high frequencies (compare Fig. 6B). (0.52 MB TIF) [file pcbi.1000768.s001.tif]

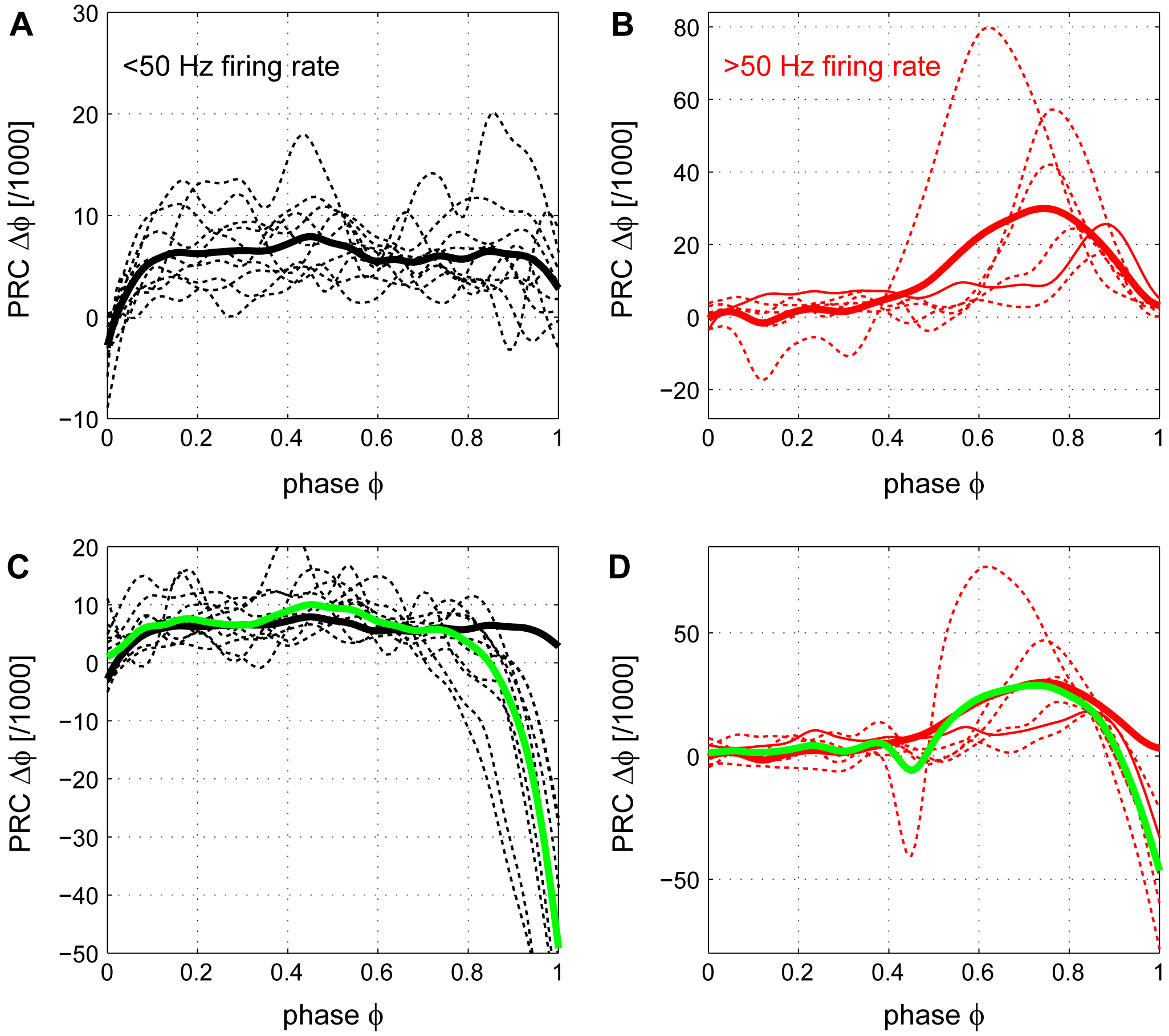

Supplement: Figure S2 — Comparison of the corrected and traditional methods for obtaining PRCs. (A) and (B) PRCs of individual Purkinje cells firing at low rates (A, black) and high rates (B, red) and population averages (thick lines), exactly as in Figure 6AB of the main manuscript. (C) and (D) PRCs of the same cells as in (A) and (B) but obtained using the traditional method. The population averages obtained with the traditional method (thick green lines) are qualitatively different from the population averages obtained using the corrected method (thick black and red lines). The bias is such that the conclusions obtained in this study would not have been possible without developing the new method. (0.32 MB TIF) [file pcbi.1000768.s002.tif]

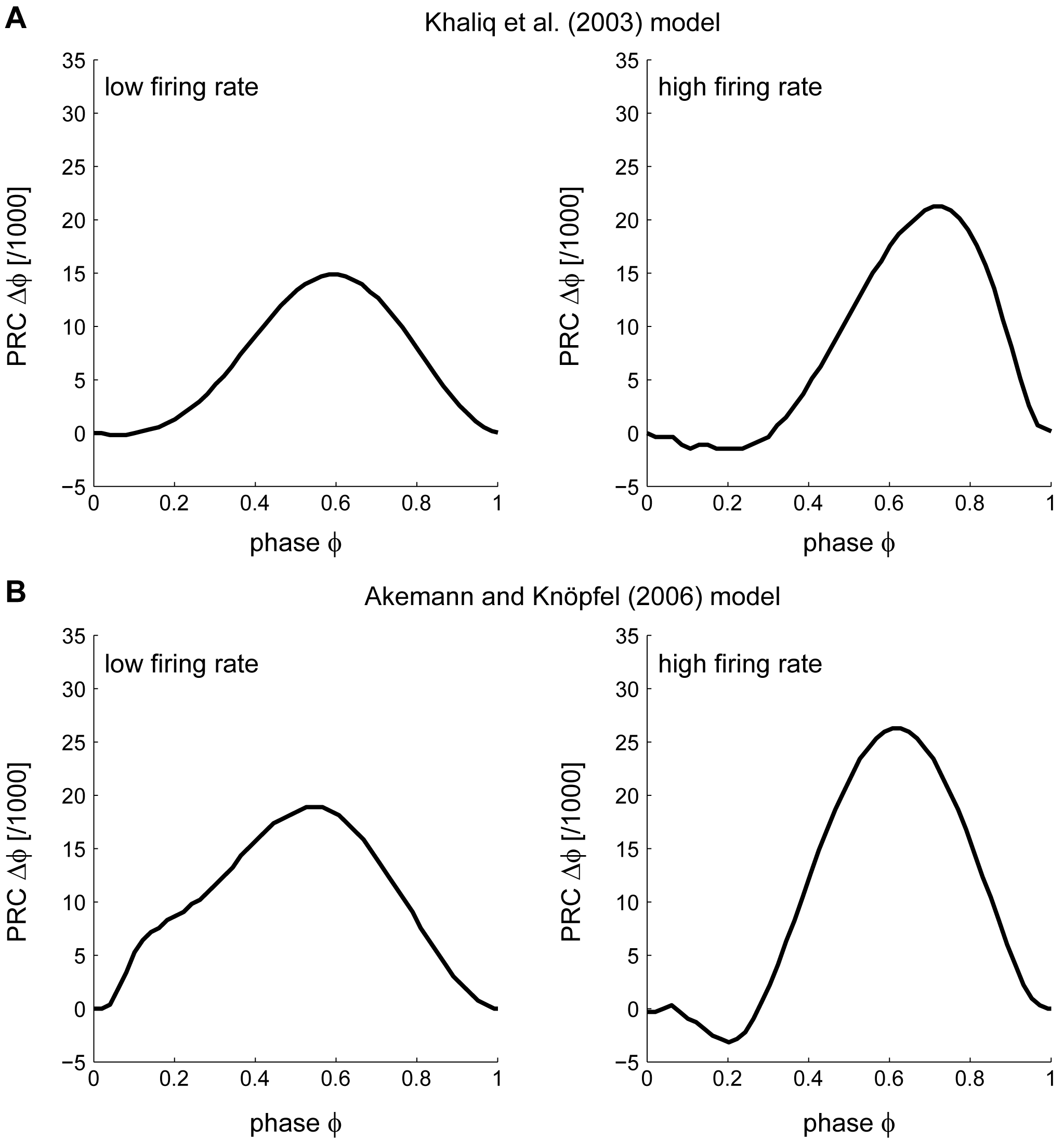

Supplement: Figure S3 — PRCs in different model neurons. (A) PRCs obtained with the model of Khaliq et al. [26] at a low firing rate (left) and a high firing rate (right) both exhibit monophasic behavior as seen in the experiment at high firing rates. (B) Frequency dependence of PRCs in an alternative Purkinje cell model from Akemann and Knöpfel [47]. A hint of a ‘shoulder’ at low firing rates indicates the trend of a frequency dependent switch in the model. However, the PRCs at low firing rates (left) are still not flat. (0.21 MB TIF) [file pcbi.1000768.s003.tif]
